# Hospital Admission Patterns in Children with CAH: Admission Rates and Adrenal Crises Decline with Age

**DOI:** 10.1155/2016/5748264

**Published:** 2016-01-06

**Authors:** R. Louise Rushworth, Henrik Falhammar, Craig F. Munns, Ann M. Maguire, David J. Torpy

**Affiliations:** ^1^School of Medicine, Sydney, The University of Notre Dame, Australia, 160 Oxford Street, Darlinghurst, NSW 2010, Australia; ^2^Department of Endocrinology, Metabolism and Diabetes, Karolinska University Hospital, 171 76 Stockholm, Sweden; ^3^Department of Molecular Medicine and Surgery, Karolinska Institutet, 171 76 Stockholm, Sweden; ^4^Menzies School of Health Research and Royal Darwin Hospital, 105 Rocklands Drive, Tiwi, NT 0810, Australia; ^5^Endocrinology and Diabetes, The Children's Hospital Westmead, Hawkesbury Road, Westmead, NSW 2145, Australia; ^6^Paediatrics and Child Health, Sydney Medical School, The University of Sydney, Sydney, NSW 2006, Australia; ^7^Endocrine and Metabolic Unit, Royal Adelaide Hospital and University of Adelaide, North Terrace, Adelaide, SA 5000, Australia

## Abstract

*Objective*. To examine patterns of hospitalisation for acute medical conditions in children with congenital adrenal hyperplasia (CAH).* Design*. A retrospective study of hospitalisation using administrative data.* Setting*. All hospitals in NSW, Australia.* Patients*. All patients admitted with CAH and a random sample of admissions in patients aged 0 to 18 years without adrenal insufficiency (AI).* Main Outcome Measures*. Admissions and comorbidities by age and sex.* Results*. Of 573 admissions for medical problems in CAH children, 286 (49.9%) were in males, and 236 (41.2%) had a principal diagnosis of CAH or had an adrenal crisis (AC). 37 (6.5%) ACs were recorded. An infection was found in 43.5% (*n* = 249) of the CAH patient admissions and 51.7% (*n* = 1613) of the non-AI group, *p* < 0.001. Children aged up to one year had the highest number of admissions (*n* = 149) and six ACs (four in males). There were 21 ACs recorded for children aged 1–5 years. Older CAH children had fewer admissions and fewer ACs. No in-hospital deaths were recorded.* Conclusions*. Admission for medical problems in CAH children declines with age. An AC was recorded in 6.5% of the admissions, with the majority of ACs occurring in the 1 to 5 years age group and there were no deaths.

## 1. Introduction

Congenital adrenal hyperplasia (CAH) is the commonest cause of adrenal insufficiency (AI) in childhood and 21-hydroxylase deficiency is the most frequently occurring form, accounting for approximately 95% of cases and having an incidence of between 1 in 9,000 and 1 in 20,000 births [1–3]. 21-Hydroxylase deficiency is autosomal recessive, with patients inheriting inactivating mutations of varying severity in both* CYP21A2* alleles, resulting in impaired synthesis and secretion of cortisol and aldosterone in combination with elevated levels of adrenal androgens [3–5]. Patients, particularly those with the more severe forms, are at risk of a salt-wasting crisis in infancy and an adrenal crisis (AC) at any age, when circulating cortisol levels are lower than physiologically required, especially in conditions of physiological stress such as infection [1, 3, 4].

Treatment of CAH involves glucocorticoid (GC) administration to provide GC replacement and to suppress ACTH stimulation, thereby reducing excess adrenal androgen production. Fludrocortisone is also used to treat aldosterone deficiency. To suppress the production of adrenal androgens, GC doses are often required in excess of physiological replacement, which may impair growth and produce manifestations of GC excess [3–7]. Importantly, affected children are also at increased risk of developing an AC, which may be fatal [4, 8–10], either due to reduced cortisol synthesis or as a result of suppression of the hypothalamic-pituitary axis due to therapeutic GC exposure. The incidence of AC in these children is approximately 4.9 per 100 patient years [8].

Neonatal screening for 21-hydroxylase deficiency by measurement of 17-hydroxyprogesterone, a steroid precursor directly proximal to the 21-hydroxylase block, aims to prevent ACs, which may be fatal, especially in males, but also in females with mild or unrecognized genital virilization [1, 3, 11]. While screening programs are established in a number of countries [12], screening is not available in Australia, nor is there a national register of patients from which health outcome data on patients affected by CAH can be extracted and analysed. Hospital administrative datasets, however, are accessible repositories of information on aspects of morbidity in these patients and are particularly useful as a source of data on serious morbidity in affected children beyond the early infant period. The aim of this study, therefore, was to provide further information on the burden of illness experienced by CAH patients by examining patterns of hospitalisation for acute medical (nonsurgical) problems in children with CAH in Australia and to identify any factors that may be associated with such hospitalisation in affected children.

## 2. Methods

New South Wales (NSW) is a region with a population of over 7 million people. All documented diseases and procedures for each patient in NSW hospitals (public and private) are coded according to the Australian modification of the International Statistical Classification of Diseases and Related Problems (diagnoses) [13] and are stored by the NSW Ministry of Health. Cases were selected from all admissions between 2001 and 2013, in patients aged up to 18 years, where the principal diagnosis (main reason for the admission) or any comorbid diagnosis was CAH (E25.0). A second random sample of all children (*n* = 3120), whose record did not contain any codes for primary or secondary adrenal insufficiency (AI) (non-AI group) and who had an admission for an acute medical problem, was selected as a comparison sample of patients. In both datasets, each record included patient age (in years); sex; principal diagnosis; all secondary diagnoses and procedures; admission to an intensive care unit (ICU); and mortality. Exclusion criteria included malignant disease, a principal diagnosis of psychiatric illness, or an obstetric or gynaecological problem; admission for rehabilitation or for planned day-only treatments or investigations such as dialysis or endoscopy; patients awaiting transfer to another hospital; admission for treatment of injuries; treatment of a surgical complication; and admissions in which a surgical procedure was performed.

An AC was identified by the code E27.2 in any diagnostic field. Infection was classified as any diagnosis caused by an organism such as a urinary tract infection (UTI), pneumonia, or where an infectious agent was coded. Specifically, a virus was identified where a viral agent was coded or where a code indicated a virus (such as viral gastroenteritis). Bacterial infections were identified in the same way. All UTIs (N39) were classified as bacterial infections. Respiratory tract illness was classified as any code relating to an acute problem in the respiratory tract.

The study was approved by the Human Resource Ethics Committee of the University of Notre Dame, Australia.

### 2.1. Statistical Analysis

Chi square and Fisher's Exact tests were used to assess the differences in frequencies of categorical variables between groups and *t*-tests were used to determine the difference in continuous variables. The data were analysed using SPSS (IBM Corp. released 2013, IBM SPSS Statistics for Windows, Version 22.0., Armonk, NY, IBM Corp.).

## 3. Results

**Figure 1:**
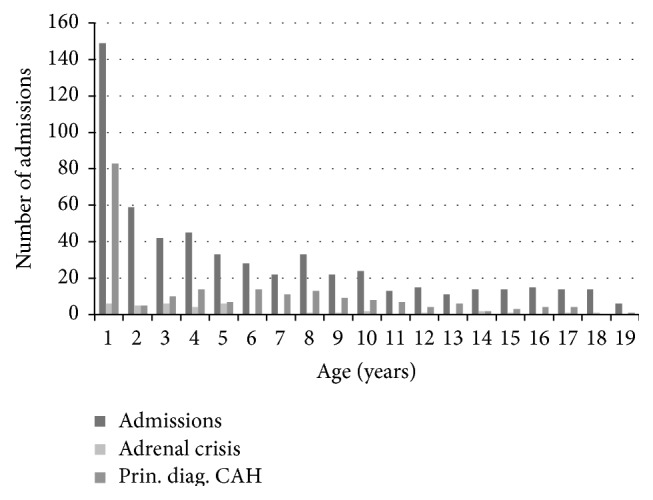
Total admissions, admissions for principal diagnosis of CAH, and admissions with an adrenal crisis in patients with CAH, NSW, 2000–2013.

During the thirteen-year study period, there were 573 admissions for acute medical problems in children and adolescents with CAH (corresponding to an average annual admission rate of 44.1 admissions/year). There were 37 (6.5%) ACs recorded. Half (*n* = 286, 49.9%) of the admissions were in males (Table 1). Overall, there were 236 (41.2%) episodes of hospitalisation for treatment of symptomatic hypoadrenalism in this group, as either a principal diagnosis of CAH (35.8%, *n* = 205) or a record of an AC (5.4%, *n* = 31) but with an alternate principal diagnosis other than CAH. Infants aged up to 12 months contributed one-quarter (26.0%, *n* = 149) of the admissions, with the number of admissions decreasing with the increasing age of the children with CAH (Figure 1). Children in the preschool (1 to 5 years) age group contributed 207 admissions (36.1%) and there were 129 (22.5%) admissions in patients aged 6 to 11 years with the remaining 15.4% (*n* = 88) aged 12 to 18 years. Infants had the highest number of admissions with a principal diagnosis of CAH (*n* = 83, 55.7%) (Figure 1). By comparison, children aged between 1 and 5 years contributed the highest number of ACs that were recorded (*n* = 21, 56.8%). No in-hospital mortality was recorded among the CAH patients.

In contrast to the CAH children, a significantly greater proportion of the random sample of non-AI children was male (*n* = 1703, 54.6%; *p* < 0.05) but the mean ages of the two groups were equal (mean (SD) CAH: 5.1 yrs (5.2), non-AI: 5.1 yrs (5.8)). The characteristics of the CAH and comparison samples are shown in Table 1. The proportion of admissions with an infection was significantly higher in the non-AI sample (51.7%, *n* = 1613) than in the CAH patient group (43.5%, *n* = 249) (*p* < 0.001). Viruses affected one-fifth of both groups but CAH patients had fewer bacterial infections than the comparison sample (CAH: *n* = 34, 5.9%; non-AI *n* = 295, 9.5%; *p* < 0.01). Respiratory tract illness and diabetes mellitus were significantly more common in the non-AI sample (both *p* < 0.001). In contrast, gastroenteritis was significantly more common among the children with CAH (*n* = 96, 16.8%) than in the comparison group (*n* = 247, 7.9%) (*p* < 0.001).

Among the admissions of infants with CAH, 52.3% (*n* = 78) were in males (Table 2). An AC was recorded in six (4.0%) admissions (four males) and a principal diagnosis of CAH was present in 55.7% (*n* = 83) of the infants, suggesting that symptomatic hypoadrenalism was the main reason for admission in more than half the children in this age group. Nearly one-fifth (*n* = 28, 18.8%) of the infants with CAH had an admission to ICU, which was significantly more than the proportion of infants in the random sample group in which there were 18 (2.2%) ICU admissions (*p* < 0.001). In addition, ICU admission was significantly more common among male than female infants in the CAH group (M: *n* = 20, 25.6%; F: *n* = 8, 11.3%; *p* < 0.05). An infection was present in a third of the admissions of infants in the CAH group (Table 2). In this age group, males had significantly more infections (M: *n* = 36, 46.2%; F: *n* = 15, 21.1%; *p* < 0.001) and respiratory tract illnesses (M: *n* = 13, 16.7%; F: *n* = 4, 5.6%; *p* < 0.05) than females.

There were twenty-one (10.1%) ACs (13 females) identified in the 207 admissions in children with CAH who were aged 1 to 5 years (Table 2). Half (50.7%, *n* = 105) of these children had an infection recorded, with 29.5% (*n* = 61) having a viral and 3.4% (*n* = 7) having a bacterial infection (Table 2). One-fifth (21.7%, *n* = 45) of the CAH children in this age group were diagnosed as having gastroenteritis (Table 2). By comparison, among the 129 children aged between 6 and 11 years, there were only 5 (3.9%) ACs (4 males) recorded (Table 2). An infection was noted in 44.2% (*n* = 57) of these children, and a viral infection (20.2%, *n* = 26) was more than three times as common as a bacterial infection (6.2%, *n* = 8).

In contrast, there were fewer patients (*n* = 88) admitted to hospital in the 12 to 18 years age group and in this group there were five ACs (5.7%) recorded (4 males) (Table 2). A diagnosis of infection was found in 40.9% (*n* = 36) of this age group, and like the situation in the younger children, viruses (18.2%, *n* = 16) were more common than bacterial (6.8%, *n* = 6) infections.

## 4. Discussion

The results of the present study, which covered thirteen years of admissions to both private and public hospitals in a large geographic area for medical treatment of children with CAH, demonstrated that infants had the highest admission rate among the age groups; episodes of hospitalisation decreased with increasing age; 6.5% of the admissions included a record of an AC but if all cases of admission for hypoadrenalism were included, almost 50% were due to some degree of AI; there was a lessening of the risk of AC beyond age of five; comorbid viral infections were more common than bacterial infections; and there were no in-hospital deaths.

Among all the CAH patient admissions, 149 were in infants, which corresponded to an average of 11.5 admissions in the infant age group for each year of the study. A pilot study of neonatal screening, conducted in the same geographic area between 1995 and 1997, found an incidence of 6 to 7 cases of classic CAH per year [2], suggesting that the infants in the present study had, at most, an average of two medical admissions each in their first year of life. Only six ACs were recorded in these children's admissions, which was lower than the number expected from studies in other unscreened populations [2, 8, 14]. However, more than half (55.7%) had a principal diagnosis of CAH, suggesting that a substantial proportion of infants in this population had symptomatic hypoadrenalism or salt-wasting coded as the reason for admission rather than an AC or that virilization was identified postnatally in an infant that had not yet exhibited salt-wasting or an AC and that this resulted in hospital admission. Nearly one-fifth (18.8%) of these infants required ICU admission, indicating that a substantial proportion of patients were significantly unwell during the admission.

Although there were slightly more admissions among male (52% of the total) than female infants, it was not possible in this analysis to assess whether the observed sex ratio reflected the true incidence of CAH. Differences in detection, morbidity at presentation (particularly the likelihood of presentation with an AC), and mortality risk between male and female infants with CAH have been identified in a number of incidence studies [1, 11, 14–17]. While there was no significant difference between the sexes in the proportion of admissions with either an AC or a principal diagnosis of CAH in this study, male infants with CAH appeared to suffer greater levels of morbidity, as they had approximately twice the number of infections, had three times the number of admissions for respiratory problems, and were twice as likely to be admitted to an ICU compared to female infants.

In this analysis, children in the one- to five-year age category had the highest proportion of ACs of the four age groups. While the reasons for the higher incidence of ACs at this age are uncertain, the children with CAH in this age group had more infections than children in other age categories, as was the case in the comparison group. Viral infections, respiratory tract illness, and gastroenteritis were the most common comorbid illnesses recorded, suggesting that management of stress dosing during intercurrent infection may have been associated with the need for parenteral GC administration and hospitalisation. The higher level of infections in both the CAH and non-AI subjects may be a reflection of an increased exposure to infection, especially viruses, as children in this age group move into childcare and start formal education, a phenomenon that may be confounded by the cessation of breastfeeding at this age, and possibly highlighting the need for increased vigilance around the management of CAH in the context of intercurrent illness at this time.

Infections are a common reason for admission to hospital in childhood, and at least one infectious illness was reported in 43.5% of all the episodes of hospitalisation for children with CAH included in this study, which was lower than the proportion of admissions with an infection in the control sample (51.7%). Infections are a known precipitant of an AC in treated AI [18, 19]. In the children with CAH, viral illnesses were much more frequent than bacterial infections, while, among adults with AI, it has been demonstrated that bacterial infections predominate [20–22]. In addition, gastroenteritis and fever are frequently identified as precipitating factors for an AC in patients with AI [18], but these were reported in only 15.7% and 3.1% of the children, respectively. Despite this, in the present study, admission with a comorbid condition of gastroenteritis was significantly more common among children with CAH than among those without AI. This is most likely due to the need for hospitalisation in children with CAH who are vomiting or have a diarrhoeal illness and who are unable to absorb appropriate GC pharmacotherapy. Patient and caregiver education about the need for stress dosing, parenteral GCs, and hospital attendance in the event of intercurrent illness are regarded as a mainstay of sick day management in AI [23]. However, recent evidence suggests that patient education does not necessarily prevent the occurrence of an AC [19].

In adolescence, the provision of clinical services to CAH patients changes, with the development of greater patient autonomy and the move from the pediatric service environment with parental oversight to an adult service [24, 25]. In this study, adolescents had the lowest annual admission rate, but more than one-quarter of the admissions were for a principal diagnosis of hypoadrenalism, either as an AC (*n* = 5) or for a principal diagnosis of CAH, indicating that severe illness related to AI remains an issue for some CAH patients into adolescence and adulthood. While adolescents with CAH who are receiving GC pharmacotherapy are at risk of an AC, there are a number of other problems that may emerge in this age group such as poor attendance at medical reviews, reduced medication adherence, and the development of testicular adrenal rest tumours in males [6, 24, 25].

The new information arising from this study illustrates the benefit of using available administrative data to evaluate the morbidity experienced by patients with chronic diseases, such as CAH. The dataset used in this analysis represents all the acute medical admissions of patients with CAH to all hospitals in a large geographic area and is not, therefore, affected by problems with selection bias and generalisability that are an issue in many studies. The analysis was enhanced by the inclusion of a randomly selected sample of admissions in children without AI. In both the CAH and non-AI groups, the clinical information that was used in the analysis represented the diagnosis conferred by the treating clinician and was obtained from the medical record. These data are subjected to regular audits at both the hospital and departmental level to ensure accuracy. By using data from all hospitals in this way, this study demonstrated the utility of such analyses in determining the burden of severe illness in rare diseases and provided information on the changes in serious morbidity experienced by children with CAH from diagnosis to the end of childhood.

However, these data also have some limitations. Both the type and severity of CAH were not captured in the dataset, nor was the exact age (in days) at presentation of infants, as the patient's age at the time of admission was included in terms of years of age. Moreover, these data are based on episodes of care rather than on individual patient histories and it is likely that some children were admitted more than once for symptomatic AI or an AC, as has been demonstrated in previous studies [8]. Further, patients in the CAH group were selected on the basis of that diagnosis appearing as a condition in the medical record, and some patient treatment episodes may have been omitted if the diagnosis was not recorded. While the data include all patients treated in NSW hospitals, it is possible that some patients, especially those living near state borders, were treated at interstate hospitals and were, therefore, not included in the analysis [26]. In addition, although there were no deaths recorded in hospital, it is possible that there were deaths that were attributable to an AC that occurred outside hospital.

In summary, the results of this population-based study suggest that AC events occur in CAH with a frequency that is up to that of AI in adults, with the 1–5-year age group at particular risk, perhaps due to frequent viral infections. It is possible that higher doses of glucocorticoids given to control androgen excess may induce secondary AI and heighten the risk of AC among some children. The low incidence of AC in infants is unexpected but may be related to differences in coding in this age group. In addition, this large study did not identify any in-hospital AC related deaths in this child/adolescent population without screening.

## Figures and Tables

**Table 1:** Demographic and disease characteristics of patients in the CAH and random sample groups, NSW, 2001–2013.

	CAH group		Random sample		*p* value
	(*n* = 573)		(*n* = 3120)		
	*n*	%	*n*	%	
Sex (male)	286	49.9	1703	54.6	<0.05
Age (mean)	5.1 (SD 5.2)		5.1 (SD 5.8)		NS
Age group (yrs)					
0	149	26.0	816	26.2	<0.001
1–5	207	36.1	1249	40.0	
6–11	129	22.5	455	14.6	
12–18	88	15.4	600	19.2	
Length of stay (days)					
1	333	58.1	1723	55.2	<0.05
2-3	140	24.4	933	29.9	
4 or more	100	17.5	464	14.9	
ICU admission	31	5.4	42	1.3	<0.001
Infection (any)	249	43.5	1613	51.7	<0.001
Bacteria	34	5.9	295	9.5	<0.01
Virus	122	21.3	632	20.3	NS
Respiratory tract illness	98	17.1	1207	38.7	<0.001
Asthma	22	3.8	422	13.5	<0.001
Bronchiolitis	13	2.3	266	8.5	<0.001
Pneumonia/LRTI	16	2.8	457	14.6	<0.001
Gastroenteritis	96	16.8	247	7.9	<0.001
UTI	15	2.6	116	3.7	NS
Fever	17	3.0	88	2.8	NS
Diabetes	2	0.3	83	2.7	<0.001

**Table 2:** Comorbid conditions in patients with CAH by age, NSW, 2001–2013.

	Up to 1 year	1–5 years	6–11 years	12–18 years
Admissions (*n*)	(149)	(207)	(129)	(88)

	*n* (%)	*n* (%)	*n* (%)	*n* (%)

Sex (males)	78 (52.3)	95 (45.9)	64 (49.6)	49 (55.7)
AC or CAH^*∗*^	85 (57.0)	69 (33.3)	57 (44.2)	25 (28.4)
Adrenal crisis	6 (3.6)	21 (9.5)	5 (3.9)	5 (5.7)
CAH^*∗*^	83 (55.7)	50 (24.2)	52 (40.3)	20 (22.7)
ICU admission	28 (18.8)	1 (0.5)	1 (0.8)	1 (1.1)
Any infection	51 (34.2)	105 (50.7)	57 (44.2)	36 (40.9)
Viral infection	19 (12.8)	61 (29.5)	26 (20.2)	16 (18.2)
Bacterial infection	13 (8.7)	7 (3.4)	8 (6.2)	6 (6.8)
Gastroenteritis	15 (10.0)	45 (21.7)	23 (17.8)	13 (14.8)
Respiratory tract illness	17 (11.4)	41 (19.8)	22 (17.1)	18 (20.5)
Asthma	—	9 (4.3)	8 (6.2)	5 (5.7)
Acute bronchiolitis	9 (6.0)	4 (1.9)	—	—
Pneumonia/LRTI^#^	7 (4.7)	8 (3.9)	1 (0.8)	—
UTI	7 (4.7)	3 (1.4)	3 (2.3)	2 (2.3)
Fever	5 (3.4)	10 (4.8)	2 (1.6)	—
Ambiguous genitalia	10 (6.7)	3 (1.4)	—	—
Fused labia	—	—	—	—
Clitoral abnormalities	2 (1.2)	—	—	—
Hypoosmolality/hyponatraemia	7 (4.7)	1 (0.5)	1 (0.8)	—
Hypoglycaemia	1 (0.7)	6 (2.9)	—	—
Hypotension	—	—	—	2 (2.3)
Hypovolaemia	9 (6.0)	15 (7.2)	9 (7.0)	3 (3.4)
Hyperkalaemia	2 (1.3)	—	—	—
Hypokalaemia	1 (0.7)	1 (0.5)	—	1 (1.1)
Nausea and vomiting	4 (2.7)	31 (15.0)	10 (7.8)	11 (12.5)
Abdominal pain	—	4 (1.9)	2 (1.6)	8 (9.1)
Syncope/collapse	—	—	—	1 (1.1)
Prematurity/neonatal problems	37 (24.8)	2 (1.0)	—	—
Hypertension	13 (8.7)	4 (1.9)	2 (1.5)	4 (4.1)
Epilepsy/convulsions	—	4 (1.9)	—	9 (9.3)
Diabetes mellitus	—	—	1 (0.8)	1 (1.0)
Obesity	—	—	—	3 (3.1)
Prec. puberty	—	2 (1.0)	3 (2.3)	3 (3.1)
Short stature	—	3 (1.4)	9 (6.8)	2 (2.1)

^*∗*^As a principal diagnosis.

^#^LRTI: lower respiratory tract infection.
